# Desires, Need, Perceptions, and Knowledge of Assisted Reproductive Technologies of HIV-Positive Women of Reproductive Age in Ontario, Canada

**DOI:** 10.5402/2012/853503

**Published:** 2012-08-16

**Authors:** Yimeng Zhang, Shari Margolese, Mark H. Yudin, Janet M. Raboud, Christina Diong, Trevor A. Hart, Heather M. Shapiro, Cliff Librach, Matt Gysler, Mona R. Loutfy

**Affiliations:** ^1^Women and HIV Research Program, Women's College Research Institute, Women's College Hospital, Toronto, ON, Canada M5G 1N6; ^2^Faculty of Medicine, University of Toronto, Toronto, ON, Canada M5S 3K1; ^3^Department of Obstetrics and Gynecology, St. Michael's Hospital, University of Toronto, Toronto, ON, Canada M5B 1W8; ^4^Clinical Decision Making and Health Care, University Health Network, Toronto, ON, Canada M5G 2C4; ^5^Dalla Lana School of Public Health, University of Toronto, Toronto, ON, Canada M5S 1A1; ^6^Department of Psychology, Ryerson University, Toronto, ON, Canada M5B 2K3; ^7^Department of Obstetrics and Gynaecology, Mount Sinai Hospital, University of Toronto, Toronto, ON, Canada M5G 1X5; ^8^Department of Obstetrics and Gynaecology, Sunnybrook Health Sciences Centre, University of Toronto, Toronto, ON, Canada M4N 3M5; ^9^Department of Obstetrics and Gynecology, Credit Valley Hospital, Mississauga, ON, Canada L5M 2N1

## Abstract

The purpose of this cross-sectional study is to assess the desire, need, perceptions, and knowledge of assisted reproductive technologies (ARTs) for women living with HIV (WLWHIV) and determine correlates of ART knowledge desire. WLWHIV of reproductive age were surveyed using the survey instrument “The HIV Pregnancy Planning Questionnaire” at HIV/AIDS service organizations across Ontario, Canada. Of our cohort of 500 WLWHIV, median age was 38, 88% were previously pregnant, 78% desired more information regarding ART, 59% were open to the idea of receiving ART, 39% felt they could access a sperm bank, and 17% had difficulties conceiving (self-reported). Age, African ethnicity, and residence in an urban center were correlated with desire for more ART information. Of participants, 50% wanted to speak to an obstetrician/gynecologist regarding pregnancy planning, and 74% regarded physicians as a main source of fertility service information. While the majority of participants in our cohort desire access to ART information, most do not perceive these services as readily accessible. Healthcare practitioners were viewed as main sources of information regarding fertility services and need to provide accurate information regarding access. Fertility service professionals need to be aware of the increasing demand for ART among WLWHIV.

## 1. Introduction

With the advent of highly active antiretroviral therapy (HAART) to treat HIV infection over the past 15 years, dramatic reductions in HIV-related morbidity and mortality have yielded improvements in quality of life and life expectancy [1]. The risk of vertical transmission through pregnancy has also been reduced to <1% with timely antiretroviral therapy, continuing viral suppression, delivery by Caesarean section if appropriate, and avoidance of breastfeeding [2]. Women now represent 22% of Canada's HIV-positive population, and with over 80% of HIV-positive women being of reproductive age [3], there is a need for safe pregnancy planning as more women living with HIV (WLWHIV) desire to become pregnant. While prevention of vertical transmission of HIV to the child remains a key component, the main focus of safe pregnancy planning includes prevention of horizontal transmission to an uninfected partner and treatment of infertility issues [4]. To achieve such objectives, assisted reproductive technologies (ARTs) have become central to meeting the reproductive needs of WLWHIV in resource-rich countries.

Indeed, access to conception and fertility services including ART has become an important component within the recent global movements to establish national guidelines or update existing recommendations on pregnancy planning and conception for people living with HIV (PLWHIV) in Canada, the United Kingdom, Germany, and France [5–11]. Furthermore, as of July 2010, the most recent American Society for Reproductive Medicine (ASRM) Ethics Committee guidelines also recommend that fertility clinics offer services to HIV-positive individuals and couples willing to use ART for reduction of transmission risk [12]. Compared to conception through unprotected intercourse with timed ovulation, associated with a horizontal transmission risk of 4.3% for serodiscordant couples [13], ART procedures such as sperm washing, intra uterine insemination (IUI), in vitro fertilization (IVF), and intracytoplasmic sperm injection (ICSI) have been found to offer significant reductions in horizontal HIV transmission or coinfection for couples wishing to conceive [13]. As well, ART is also recommended for the treatment of infertility, an issue of importance to WLWHIV as studies have indicated lower fertility rates among HIV-positive women [14–22]. Various studies have documented an increased rate of tubal factor infertility in HIV-positive women [7, 14, 15, 23].

Previous literature on the fertility intentions of WLWHIV has been important in eliciting trends and predictors of the decision to attempt pregnancy [24–39]. As well, studies exist regarding the prevalence of fertility service centers in North America receiving requests from or providing services to PLWHIV [6, 12, 31]. Although the ASRM guidelines recommend that fertility clinics offer reproductive assistance to HIV-positive patients within technical and economic feasibility, less than 3% of fertility practices registered with the American Society for Assisted Reproductive Technologies provide services to couples where both partners are HIV positive [12, 31]. Low patient volume has been cited as the most common reason for the limitation of fertility services by American service providers [31]. Few studies have investigated the demand for or desire to use ART or the trends in and predictors of conception strategies amongst PLWHIV. As women are the main recipients of ART, it is important to assess the current intentions, knowledge, and perceptions regarding ART among the current cohort of WLWHIV in North America. The purpose of this cross-sectional study was to assess the desire and need for ART among HIV-positive women of reproductive age living in Ontario, Canada, and to determine correlates of ART knowledge desire and the knowledge and perceptions of accessibility of ART by the WLWHIV in this cohort.

## 2. Materials and Methods

### 2.1. Study Design and Population

A cross-sectional study using a survey instrument was carried out with participants who met the following inclusion criteria: (1) HIV-positive, (2) biologically female, (3) of reproductive age (ages 18–52), (4) living in Ontario, Canada, and (5) ability to read English or French. The upper age limit was chosen to reflect the cut-off for fertility clinic consultation in Canada. The details of the original cross-sectional study have been previously described [40].

### 2.2. Recruitment and Ethics

 Recruitment was conducted from October 5th, 2007 to March 31st, 2009 through 28 AIDS service organizations (ASOs), eight primary care and specialty HIV clinics, and two community health centers (CHCs) across the province of Ontario. An invitation email was sent out to all Ontario ASOs listed on the Canadian AIDS Society website (the national society for ASOs) and all clinics and CHCs known by the investigators that care for HIV-positive women. Recruitment and study qualification determination were carried out by a single research staff member at each site following a predeveloped recruitment plan (available upon request) [40]. The recruitment was carried out in a consecutive manner. Our study determined geographic distribution of our data collection sites based on the provincial regions laid out by the provincial public health authorities with the specific regions noted in Table 1. This nonrandom sampling technique was used in an effort to obtain a representative sample of HIV-positive women of reproductive age living in Ontario in the absence of a registry of HIV-positive individuals. Once written consent was obtained, the survey was completed at the site or at home.

 Research Ethics Board approval was obtained prior to study initiation. Written informed consent was obtained from every participant. An amended ethics approval was obtained for this specific research analysis.

### 2.3. Survey Instrument and Validation

 A 189-item survey instrument, “The HIV Pregnancy Planning Questionnaire,” was created using the methods of Fowler for instrument development [41] and has previously been described in detail [40]. The survey consisted of 12 domains including: (1) interest/desire to have children, (2) intent to have children in the future, (3) behavior related to the pursuit of fertility, (4) menstrual, birth control, and sexual history, (5) pregnancy and birth history, (6) perceived support for becoming pregnant, (7) satisfaction with providers, (8) needs assessment, (9) HIV medical history, (10) demographics, (11) anxiety and depression, and (12) HIV stigma (full survey instrument available upon request).

 The survey was first developed in English and then translated into French using the back translation method [40]. Content validity was achieved by using items from 4 previously validated surveys [13, 24, 32, 42], by developing items based on a literature search exploring factors that determined reproductive decisions in HIV-positive women (9–39) and by reviewing the questions with a project advisory panel of experts including HIV specialists, obstetricians, midwives, community members, and PLWHIV. Face validity was achieved by initially piloting the survey with 20 HIV-positive women in 2 focus groups. The focus group participants confirmed that the survey instrument items actually appeared to be measuring the intended items related to the domains. Furthermore, participants were asked to comment on each item in terms of comprehension, clarity, and relevance. Further pilot testing was carried out with an initial 52 HIV-positive women that met the inclusion criteria.

### 2.4. Statistical Analysis

Baseline characteristics of the study population were summarized using medians and interquartile ranges (IQRs) for continuous variables and frequencies and proportions for categorical variables. The geographic distribution of the study population was compared to the distribution of HIV-positive women living in Ontario using the Chi-square test.

The primary outcome of interest for this analysis was desire for information about ART (ART knowledge desire). The question used to represent desire for ART information was “I would like to learn more about fertility technologies and options for people living with HIV.” The response options characterized agreement with the statement on a Likert-type scale. Participant responses were dichotomized into “Yes” or “No” with regard to desire for ART. Participant answers of “Strongly agree” or “Agree” were classified as “Yes”; participant answers of “Strongly disagree” or “Disagree” or “Neither” were classified as “No”. The dichotomized participant responses were compared across the various demographic variables. The secondary outcome of interest for this analysis was past need for ART. The question used to represent previous need for ART was “If you have been pregnant before, did you ever experience problems or difficulties in trying to get pregnant?” Women who had never been pregnant and women who had been pregnant but did not answer this question were excluded from the analysis. Participant responses were compared across the various demographic variables. Univariate logistic regression analysis was conducted to determine the unadjusted odds ratios with 95% CIs for correlates of the desire for ART and need for ART. The final multivariate logistic regression model determined the adjusted odds ratios with 95% confidence intervals (CIs) for correlates of the desire for ART and need for ART. The final multivariable logistic regression models included covariates that were a priori believed to be related to desire for ART and need for ART, such as age, ethnic background, marital status, history of HIV medication, and current contraceptive use. Variables which were significant at *P* < 0.20 in the univariate analyses were candidates for inclusion in final multivariable logistic regression models for desire and need for ART. When multiple covariates measured similar phenomenon (e.g., ethnic background and country of birth), the variable representing each construct with the most statistical significance was chosen. Finally, the qualifying variables were entered into the multivariable model and selected by stepwise-selection method. Only the correlates with a significant effect (*P* < 0.05) remained in the final multivariable models.

Other outcomes of interest included perceptions and knowledge of ART. The outcomes were reported using medians and IQR for continuous variables and proportions for categorical variables. Survey data was entered twice and verified prior to analysis. Statistical analyses were performed using SAS Version 9.2 (SAS Institute, Cary, NC, USA).

## 3. Results

### 3.1. Study Population

Five hundred and four WLWHIV in Ontario, Canada, were recruited from 38 sites. Of these participants, four did not meet the inclusion criteria (two were over the age of 52 and two were not living in Ontario). Of the remaining 500 participants, 10 did not answer the question used to represent desire for ART. Surveys from the remaining 490 participants were used for the final analysis for the primary outcome.

The median age for this final study population was 38 years (IQR 32, 43). Sixty-one percent of the participants were born outside of Canada and 46% were of African ethnicity. Within this cohort, 55% were in sexual relationships and 46% were married, common-law, or living with a partner. Thirty-three percent of the women had HIV-negative partners and 24% had HIV-positive partners; 36% were currently using contraception; 88% had been pregnant previously; 74% had given birth. The demographic characteristics of the entire study population are presented in Table 1.

### 3.2. ART Knowledge Desire

In the final study population of 490 HIV-positive women living in Ontario, Canada, 78% (*n* = 384) indicated that they would like to learn more about fertility technology options. The distribution of participant characteristics by desire for ART information is shown in the logistic regression models for correlates of desire for ART in Table 2. In the final multivariate model, African ethnicity (OR = 3.86, *P* < 0.0001) and current contraceptive use (OR = 1.70, *P* = 0.05) were associated with a desire for ART. Being married, common-law, or living with a partner (OR = 0.52, *P* = 0.04) and history of HIV medication (OR = 0.34, *P* = 0.02) were associated with a lack of desire for ART.

### 3.3. HIV-Positive Women's Need for ART

Study participants were also assessed for previous need for ART. Of the 500 participants who met the study inclusion criteria, 65 did not answer the question used to represent need for ART and 10 had never been pregnant. The final analysis included surveys from 425 participants.

The distribution of participant characteristics by need for ART is shown in the logistic regression models for correlates of need for ART in Table 3. For the study participants, 17% (*n* = 72) indicated they had difficulties getting pregnant. In the final multivariate model, annual household income of $20,000 to $40,000 (OR = 2.36, *P* = 0.02) greater than $40,000 (OR = 2.63, *P* = 0.01) and current HIV treatment (OR = 2.53, *P* = 0.01) were associated with difficulties conceiving and two or more lifetime pregnancies (OR = 0.41, *P* < 0.01) were associated with no difficulties conceiving.

### 3.4. Perceptions and Knowledge of ART

Study participants were also assessed for perceptions towards and knowledge of ART. The final analysis included surveys from the 500 participants who met the study inclusion criteria and responded to these set of questions. The distribution of participant answers to the various questions representing perceptions towards and knowledge of ART is shown in Table 4.

 The majority of participants (59%) were open to medical techniques that help with conception and 39% felt they could access a sperm bank if needed. Of the women surveyed, 74% agreed they could ask a physician if they needed help finding a fertility clinic. To help with the decision to become pregnant, 50% of the participants indicated they wanted information booklets and publications on becoming pregnant, 59% wanted to speak to a health professional with fertility expertise, and 50% wanted to speak to an obstetrician/gynecologist.

## 4. Discussion

In this study, a large majority of WLWHIV (78%) indicated a desire for information regarding reproductive technology options. Correlates of desire for ART, a measure of pregnancy intention, included younger age, ethnicity, residence in an urban region, and birth place outside of Canada. Previous publications support our findings of younger age and Black/African ethnicity as significant correlates of pregnancy intention in WLWHIV [26, 28, 29, 34, 35, 39, 43, 44]. Although African ethnicity has not been associated with desire to have children, women of African ethnicity may be more likely to intend pregnancy due to cultural factors and traditional gender roles that stress the importance of childbearing [44–47]. History of HIV medication use was also associated with a lack of desire for ART in our study. However, other studies have indicated a positive influence on childbearing decisions associated with HAART [27, 30, 32, 33, 35]. These other studies, conducted in Vietnam, India, and South Africa, may have reflected the more advanced disease of the study populations, where HAART access is associated with better health and greater perceived ability to be a fit parent. In direct contrast to a previous study in British Columbia (BC), Canada, being in a stable relationship was associated with a lack of desire for ART in our study. This discrepancy can be attributed to the epidemiology of HIV infection in BC, differences in sample characteristics of the study, and the location and timing of the recruitment process [28]. These differences highlight potential regional differences in the reproductive concerns of WLWHIV, attitudes towards those seeking ART, and a need for continued research in this area.

In addition to desire, our study also investigated potential need for reproductive technology options among WLWHIV by assessing correlates of self-reported conception difficulty. Although not a direct measure of the true infertility rates among WLWHIV, conception difficulty does provide an estimate of the prevalence of infertility concerns within the population. Infertility, defined as inability to conceive 12 months after initiating attempts, affects 10–15% of the general population [48]. Various previous studies have documented the lower fertility rates of WLWHIV [14–23] and the percentage of WLWHIV who reported difficulties conceiving in our study (17%) was higher than that found in general population. This percentage is higher even though the primary infertility of our participants did resolve. WLWHIV with resolved primary infertility are not reflective of the population of WLWHIV and our results may underestimate the true infertility rates of WLWHIV. It appears then that the potential need for ART among WLWHIV may be higher than that of the general population. Greater household income and current HIV treatment were both correlates of difficulty conceiving, potentially due to the association between higher income and age. However, age was not a correlate of conception difficulty in our study. The lack of association between age, a major predictor of infertility generally, and difficulty conceiving in this study may be attributed to the relative youth of the study population. The age range of study participants was 32–43, and previous attempts at pregnancy may have occurred when the participants were younger (<35 years) when age did not significantly affect fertility. Current HIV treatment, while associated with increased fertility in developing countries [27, 30, 32, 33, 35], was associated with difficulties conceiving in our study. Thus far, studies on the effects of HAART on pregnancy rates have not shown conclusive evidence of any impact on fertility [49–51]. More research is required to further evaluate the true effects of HAART on the fertility of WLWHIV.

This study also assessed the perceptions and knowledge of WLWHIV of ART. In our study, at least half of the participants viewed information booklets and publications on becoming pregnant, speaking with a health care professional with fertility expertise, or speaking with a obstetrician/gynecologist as important resources needed to help with the decision to become pregnant. More than one-third of participants also indicated a desire to have access to a fertility clinic as a resource needed for making pregnancy decisions. The majority of women surveyed were open to receiving ART but only 39% of participants believed they had access to ART services such as sperm banks. It appears that although women are interested in pursuing ART services, most do not perceive these services as being accessible. Since 74% of study participants indicated they regard their physician as the first source of information for ART, doctors therefore have an important role in informing their patients of their full reproductive options. With the current movement towards increasing provision of ART to PLWHIV for prevention of horizontal transmission and treatment of subfertility, there is an increased need for physicians to provide information and initiate discussions regarding pregnancy planning and conception to their patients who are HIV positive.

The assessment of ART desires of a cohort of WLWHIV in Ontario, Canada, has important implications for healthcare providers, policy makers, and researchers. The results of this survey indicate WLWHIV of reproductive age are open to and desire access to ART for the purposes of safe conception and treatment of infertility. While this desire and demand for fertility technology options is supported by ASRM Ethics Committee [12] guidelines for safe pregnancy planning in North America, access to fertility services remains a limiting factor in the provision of reproductive care for WLWHIV. Although the majority of fertility clinics surveyed across Canada and the US have expressed willingness to provide care to HIV-positive individuals, less than 50% have actually offered treatment [31, 52]. Even though previous restrictive state laws that have explicitly limited the availability of ART services to PLWHIV [13] did contribute to the lack of access to fertility clinics within the US, low patient volume is the most common reason given by clinics for not providing services [31]. The perceived inaccessibility of these services by PLWHIV may contribute to the low number of clinics who actually treat patients from this population. There is a need for both provider and patient education to ensure the necessary provision of safe reproductive services to HIV-positive individuals. Healthcare practitioners need to provide accurate information regarding reproductive technology options and access to such services, while fertility service professionals need to be aware of the increasing demand for ART among PLWHIV and current recommendations regarding provision of treatment.

The limitations of our study include a potential for selection bias, as the survey used for this study was provided only in English and French and potentially limited to women literate in these two languages. As the survey was self-administered to ensure privacy, the lack of added clarification by an interviewer may have contributed to certain questions being unanswered. The participants of this study were also relatively healthy, with high CD4 counts and good viral suppression on HAART, and may not represent the full spectrum of WLWHIV in Ontario. Finally, women who desire and intend pregnancy may be more motivated to complete a survey assessing reproductive intention. However, the large sample size in the study and the matching of study recruitment to the geographic distribution of HIV-positive women living in Ontario allowed for generalizability of study results to the population of WLWHIV in Ontario. A significant proportion of the study enrolment was also conducted using a community-based research model involving AIDS Service Organizations and WLWHIV in the recruitment coordination and process, which allowed the engagement of participants who do not usually partake in research.

Our findings indicate WLWHIV of reproductive age living in Ontario, Canada both desire and need to receive ART for safe pregnancy planning and conception and view healthcare providers as the main source of information regarding reproductive options. This study continues a series of assessments intended to guide the development of Canadian national guidelines on safer pregnancy planning and conception, as well as provincial and national HIV pregnancy planning and fertility programs. Potential future areas of research include an exploration of healthcare provider attitudes towards fertility and pregnancy for HIV-positive individuals as well as exploration of potential regional differences in their reproductive needs. To fully assess the reproductive needs of PLWHIV, similar research is also being considered for the population of men living with HIV.

## Figures and Tables

**Table 1:** Demographic characteristics of study participants.

Characteristics	*N* = 490
Age (years): median (IQR)	38 (32, 43)
18–25	28 (6%)
26–40	269 (57%)
>40	179 (38%)
Ethnic background: *N* (%)	
African	217 (46%)
Caribbean	56 (12%)
European-British	57 (12%)
French-Canadian	52 (11%)
Aboriginal	40 (9%)
Other	48 (10%)
Birth place: *N* (%)	
Africa	209 (44%)
Canada	187 (39%)
Caribbean	49 (10%)
Other	32 (7%)
Not Born in Canada: *N* (%)	290 (61%)
Years in Canada: median (IQR)	5 (2, 13)
Region in Ontario: *N* (%)	
Toronto	253 (52%)
Ottawa	64 (13%)
Other	173 (35%)
Sexual orientation: *N* (%)	
Heterosexual	408 (89%)
Lesbian/Bisexual	43 (9%)
Other	8 (2%)
Work*: N* (%)	
Working	184 (39%)
On government assistance	235 (49%)
Marital status: *N* (%)	
Married/common-law/living with a partner	210 (46%)
Divorced/widowed	113 (25%)
Never married	135 (29%)
Education: *N* (%)	
High school or higher	296 (69%)
Less than high school	133 (31%)
Annual household income: *N* (%)	
<20 K	189 (46%)
20–40 K	124 (30%)
>40 K	94 (23%)
Clinical characteristics	
Duration of HIV diagnosis (years): median (IQR)	7.0 (4.0, 12.0)
Hepatitis B coinfected: *N* (%)	16 (3%)
Hepatitis C co-infected: *N* (%)	67 (14%)
Recent CD4 count: median (IQR)	466 (320, 685)
≥200 cells/mm^3^: *N* (%)	315 (90%)
Recent VL(log_10_ copies/mL): median (IQR)	3.8 (2.9, 4.5)
Ever on HIV Medication: *N* (%)	414 (86%)
Currently on HIV treatment: *N* (%)	356 (74%)
Years on treatment: median (IQR)	4.5 (1.9, 9.1)
Sexual history	
Partner: *N* (%)	
HIV negative	162 (33%)
HIV positive	118 (24%)
No partner	160 (33%)
Unknown	44 (9%)
In sexual relationship: *N* (%)	265 (55%)
Monogamous relations: *N* (%)	250 (51%)
Current contraceptive use: *N* (%)	172 (36%)
Fertility history: *N* (%)	
Ever had pregnancies	422 (88%)
Ever gave birth	358 (74%)

IDU: injection drug use; VL: viral load.

**Table 2:** Univariate and multivariate logistic regression models of participant responses for desire for ART information.

Characteristics	ART intentions		Unadjusted		Adjusted	
	Yes (*n* = 384)	No (*n* = 106)	Odds ratio (95% CIs)	*P* value	Odds ratio (95% CIs)	*P* value
Age (years):						
18–25	20 (5%)	8 (8%)	1		1	
26–40	231 (62%)	38 (36%)	2.43 (0.99, 5.91)	0.05	2.51 (0.93, 6.75)	0.07
>40	120 (32%)	59 (56%)	0.81 (0.34, 1.96)	0.64	0.78 (0.29, 2.11)	0.63
Ethnic background						
African	197 (53%)	20 (20%)	4.47 (2.63, 7.61)	<0.0001	3.86 (2.16, 6.92)	<0.0001
Other	174 (47%)	79 (80%)	1		1	
Birth place						
Africa	187 (50%)	22 (21%)	1			
Canada	126 (34%)	61 (58%)	0.24 (0.14, 0.42)	<0.0001		
Caribbean	36 (10%)	13 (12%)	0.33 (0.15, 0.71)	<0.01		
Other	23 (6%)	9 (9%)	0.30 (0.12, 0.73)	<0.01		
Not born in Canada	246 (64%)	44 (42%)	2.51 (1.62, 3.90)	<0.0001		
Years in Canada (for those not born in Canada)	4.8 (2.0, 11.0)	10.0 (3.3, 25.0)	0.95 (0.92, 0.97)	<0.001		
Toronto region	210 (55%)	43 (41%)	1.77 (1.14, 2.74)	0.01		
Working	142 (38%)	42 (42%)	0.86 (0.55, 1.34)	0.51		
On government assistance	189 (51%)	46 (46%)	1.22 (0.79, 1.90)	0.37		
Marital status						
Married/common-law/living with a partner	154 (43%)	56 (57%)	0.48 (0.27, 0.84)	0.01	0.52 (0.27, 0.97)	0.04
Divorced/widowed	91 (25%)	22 (22%)	0.72 (0.37, 1.40)	0.33	0.84 (0.39, 1.79)	0.64
Never married	115 (32%)	20 (20%)	1		1	
Education						
High school or higher	239 (71%)	57 (63%)	1.44 (0.89, 2.34)	0.14		
Less than high school	99 (29%)	34 (37%)	1			
Annual household income						
<20 K	149 (47%)	40 (44%)	1			
20–40 K	107 (34%)	17 (19%)	1.69 (0.91, 3.14)	0.10		
>40 K	60 (19%)	34 (37%)	0.47 (0.27, 0.82)	<0.01		
Years since diagnosis of HIV positive	6 (3, 11)	10 (5, 16)	0.93 (0.89, 0.97)	<0.001		
Hepatitis B co-infected	16 (4%)	0 (0%)	§			
Hepatitis C co-infected	52 (14%)	15 (15%)	0.96 (0.52, 1.79)	0.91		
CD4 ≥ 200 cells/mm^3^	248 (91%)	67 (88%)	1.28 (0.57, 2.86)	0.55		
Recent VL (log_10_ copies/mL)	3.8 (2.9, 4.6)	3.9 (2.8, 4.5)	0.93 (0.55, 1.59)	0.80		
Ever on HIV medication	315 (83%)	99 (94%)	0.30 (0.13, 0.72)	<0.01	0.34 (0.14, 0.85)	0.02
Currently on HIV treatment	276 (74%)	80 (77%)	0.84 (0.50, 1.39)	0.49		
Partner						
HIV negative	125 (58%)	37 (59%)	1			
HIV positive	92 (42%)	26 (41%)	1.05 (0.59, 1.85)	0.87		
In sexual relationship	205 (54%)	60 (57%)	0.88 (0.57, 1.37)	0.58		
Monogamous relations	193 (51%)	57 (54%)	0.86 (0.56, 1.33)	0.51		
Current contraceptive use	124 (33%)	48 (45%)	0.60 (0.39, 0.93)	0.02	1.70 (1.00, 2.88)	0.05
Fertility history:						
Ever had pregnancies	327 (87%)	95 (90%)	0.70 (0.34, 1.44)	0.33		
Ever gave birth	279 (74%)	79 (75%)	0.95 (0.57, 1.56)	0.83		

^§^Odds ratio not available. IDU: injection drug use; VL: viral load.

**Table 3:** Univariate and multivariate logistic regression models of participant responses for need for ART.

Characteristics	Problems becoming pregnant		Unadjusted		Adjusted	
	Yes (*n* = 72)	No (*n* = 353)	Odds ratio (95% CIs)	*P* value	Odds ratio (95% CIs)	*P* value
Age (years)	38 (33, 43)	38 (33, 44)				
18–25	2 (3%)	18 (5%)	1		1	
26–40	44 (64%)	181 (53%)	2.19 (0.49, 9.78)	0.31	1.60 (0.33, 7.69)	0.56
>40	23 (33%)	144 (42%)	1.44 (0.31, 6.61)	0.64	1.13 (0.23, 5.59)	0.88
Ethnic background						
African	25 (35%)	168 (50%)	1			
Caribbean	7 (10%)	33 (10%)	1.43 (0.57, 3.57)	0.45		
European-British	10 (14%)	33 (10%)	2.04 (0.89, 4.64)	0.09		
French-Canadian	11 (15%)	38 (11%)	1.95 (0.88, 4.29)	0.10		
Aboriginal	8 (11%)	30 (9%)	1.79 (0.74, 4.35)	0.20		
Other	10 (14%)	35 (10%)	1.92 (0.85, 4.35)	0.12		
African ethnicity versus other			0.55 (0.32, 0.93)	0.03		
Birth place						
Africa	26 (37%)	161 (47%)	1			
Canada	34 (48%)	126 (37%)	1.67 (0.95, 2.93)	0.07		
Caribbean	7 (10%)	30 (9%)	1.44 (0.58, 3.63)	0.43		
Other	4 (6%)	25 (7%)	0.99 (0.32, 3.08)	0.99		
Not born in Canada	37 (51%)	216 (61%)	0.67 (0.40, 1.12)	0.12		
Years in Canada (for those not born in Canada)	8 (3, 17)	5 (2, 11)	1.03 (0.99, 1.06)	0.13		
Toronto region	33 (46%)	181 (51%)	0.80 (0.48, 1.34)	0.40		
Working	27 (39%)	129 (38%)	1.04 (0.61, 1.77)	0.88		
On government assistance	36 (51%)	172 (50%)	1.05 (0.63, 1.76)	0.84		
Marital status						
Married/common-law/living with a partner	34 (50%)	152 (46%)	1.08 (0.57, 2.05)	0.82		
Divorced/widowed	17 (25%)	96 (29%)	0.85 (0.41, 1.78)	0.67		
Never married	17 (25%)	82 (25%)	1			
Education						
High school or higher	43 (69%)	204 (66%)	1.14 (0.63, 2.06)	0.66		
Less than high school	19 (31%)	103 (34%)	1			
Annual household income						
<20 K	19 (30%)	150 (51%)	1		1	
20–40 K	23 (37%)	85 (29%)	2.14 (1.10, 4.15)	0.02	2.36 (1.16, 4.80)	0.02
>40 K	21 (33%)	60 (20%)	2.76 (1.39, 5.50)	<0.01	2.63 (1.25, 5.52)	0.01
Years since diagnosis of HIV positive	9 (4, 13)	7 (4, 12)	1.00 (0.99, 1.01)	0.74		
Hepatitis B co-infection	3 (4%)	9 (3%)	1.60 (0.42, 6.08)	0.49		
Hepatitis C co-infection	11 (15%)	54 (16%)	0.96 (0.47, 1.94)	0.90		
CD4 ≥ 200 cells/mm^3^	55 (92%)	220 (90%)	1.25 (0.46, 3.41)	0.66		
Recent VL (log_10_ copies/mL)	4 (4, 5)	4 (3, 4)	1.90 (0.91, 3.97)	0.09		
Ever on HIV medication	67 (93%)	297 (85%)	2.30 (0.88, 5.98)	0.09		
Currently on HIV treatment	62 (86%)	248 (72%)	2.40 (1.18, 4.87)	0.02	2.53 (1.12, 5.71)	0.03
Partner						
HIV negative	27 (66%)	105 (53%)	1			
HIV positive	14 (34%)	92 (47%)	0.59 (0.29, 1.20)	0.14		
In sexual relationship	37 (52%)	190 (54%)	0.91 (0.55, 1.52)	0.72		
Monogamous relations	35 (49%)	181 (52%)	0.91 (0.55, 1.52)	0.73		
Current contraceptive use	30 (42%)	115 (33%)	1.43 (0.85, 2.41)	0.17		
Ever gave birth	56 (79%)	304 (87%)	0.55 (0.29, 1.06)	0.07		
Lifetime pregnancies						
At least once	23 (33%)	61 (17%)	1		1	
≥2	47 (67%)	288 (83%)	0.43 (0.24, 0.77)	<0.01	0.41 (0.21, 0.78)	<0.01
Lifetime births						
At least once	45 (63%)	142 (41%)	1			
≥2	26 (37%)	207 (59%)	0.40 (0.23, 0.67)	<0.001		

VL: viral load.

**Table 4:** Participant responses to questions related to perception of and knowledge of ART.

Perception of ART	Total (*n* = 500)
Open to medical techniques	
Strongly agree/agree	285 (59%)
Neither	68 (14%)
Strongly disagree/disagree	133 (27%)
Can go to sperm bank	
Strongly agree/agree	192 (39%)
Neither	148 (30%)
Strongly disagree/disagree	153 (31%)
Have tried to access sperm from a sperm bank	8 (2%)

Knowledge of ART	Total (*n* = 500)

Important sources of information regarding ART:	
A friend	73 (15%)
A family member	56 (12%)
A physician	357 (74%)
Other health care professionals	232 (48%)
Nobody	48 (10%)
Resources needed to help with decision to become pregnant:	
Information booklets and publications on becoming pregnant	213 (50%)
To talk to a health professional with fertility expertise	254 (59%)
To talk someone with fertility expertise from my community	113 (26%)
Access to a fertility clinic	155 (36%)
An obstetrician/gynecologist	213 (50%)
A trained midwife	72 (17%)
Access to sperm banks	84 (20%)
Educational seminars on pregnancy	138 (32%)
Educational seminars on taking care of a baby	100 (23%)
Educational seminars on raising a child	102 (24%)
